# Putting Culture Under the *‘Spotlight’* Reveals Universal Information Use for Face Recognition

**DOI:** 10.1371/journal.pone.0009708

**Published:** 2010-03-18

**Authors:** Roberto Caldara, Xinyue Zhou, Sébastien Miellet

**Affiliations:** 1 Department of Psychology and Centre for Cognitive Neuroimaging, University of Glasgow, Glasgow, United Kingdom; 2 Department of Psychology, Sun Yat-Sen University, Guangzhou, China; Kyushu University, Japan

## Abstract

**Background:**

Eye movement strategies employed by humans to identify conspecifics are not universal. Westerners predominantly fixate the eyes during face recognition, whereas Easterners more the nose region, yet recognition accuracy is comparable. However, natural fixations do not unequivocally represent information extraction. So the question of whether humans universally use identical facial information to recognize faces remains unresolved.

**Methodology/Principal Findings:**

We monitored eye movements during face recognition of Western Caucasian (WC) and East Asian (EA) observers with a novel technique in face recognition that parametrically restricts information outside central vision. We used *‘Spotlights’* with Gaussian apertures of 2°, 5° or 8° dynamically centered on observers' fixations. Strikingly, in constrained *Spotlight* conditions (2° and 5°) observers of both cultures *actively* fixated the same facial information: the eyes and mouth. When information from both eyes and mouth was simultaneously available when fixating the nose (8°), as expected EA observers shifted their fixations towards this region.

**Conclusions/Significance:**

Social experience and cultural factors shape the *strategies* used to extract information from faces, but these results suggest that external forces do not modulate *information* use. Human beings rely on identical facial information to recognize conspecifics, a universal law that might be dictated by the evolutionary constraints of nature and not nurture.

## Introduction

As noted by Galton over one century ago [1], the human capacity for face recognition is remarkable compared to the recognition of other objects. This critical biological function is a basic requirement for efficient social interactions, for all humans within all cultures. We recently questioned the universality of how face recognition is achieved by recording eye movements [2]. We showed that Westerners predominantly fixate the eye region to learn and recognize faces, a well established finding in the eye movement literature on faces [3], [4], [5], [6], [7], [8], [9]. However, contrary to all prior knowledge, Easterners consistently focus more on the nose, yet recognition accuracy was comparable. Such cultural diversity in eye movements was robust over time and generalized across different face processing tasks (learning, recognition and categorization by race).

**Figure 1:**
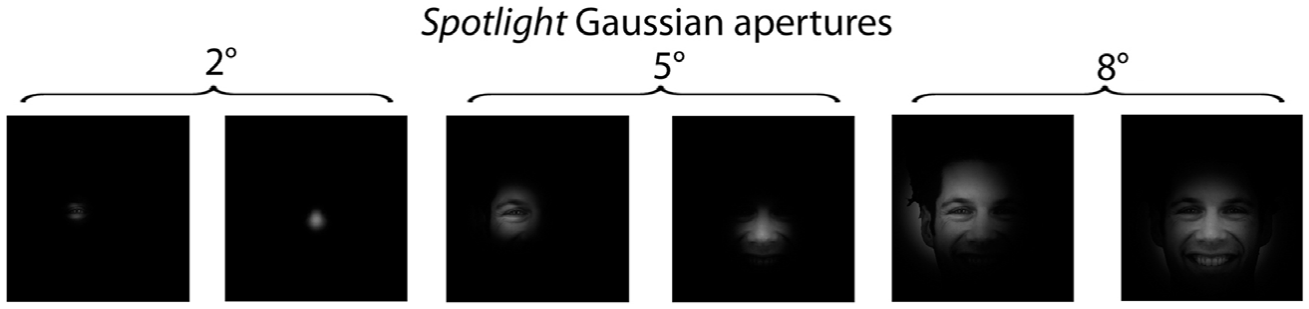
Area covered by *Spotlights* with Gaussian apertures of 2°, 5° and 8°, centered respectively on the left eye and the nose region. Note that information from both eyes and the mouth is available from the nose region only in the 8° condition.

**Figure 2:**
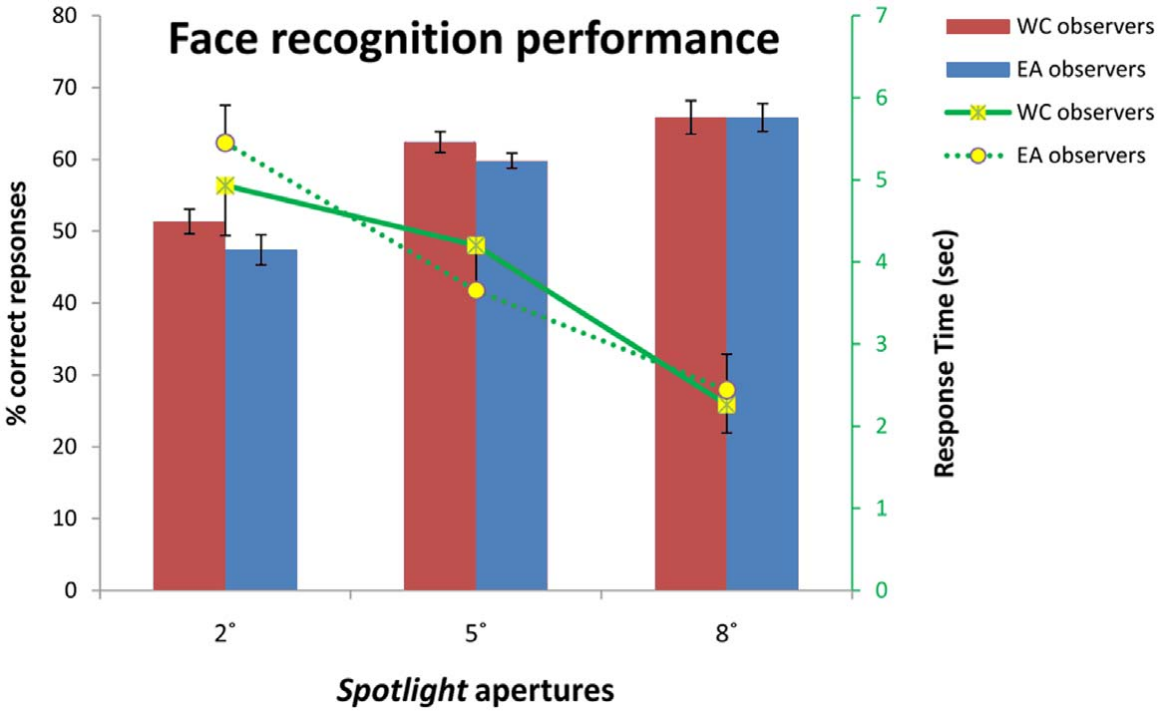
Percentages of correct responses (left *y* axis) and response times (right *y* axis) for the different *Spotlight* conditions. WC  =  Western Caucasian; EA  =  East Asian. Note the relationship for those variables, the increase of accuracy with larger *Spotlight* apertures was also accompanied by faster response times (WC: plain lines; EA: dashed lines).

**Figure 3:**
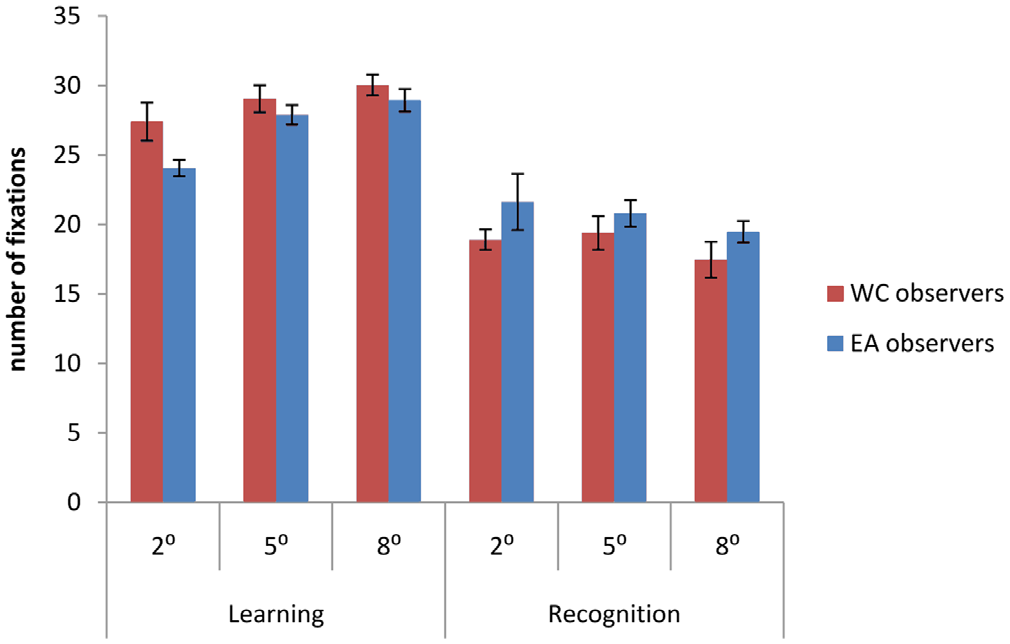
Number of average fixations employed by Western Caucasian (WC) and East Asian (EA) observers to adapt to face learning and recognition across the different *Spotlight* apertures sizes.

**Figure 4:**
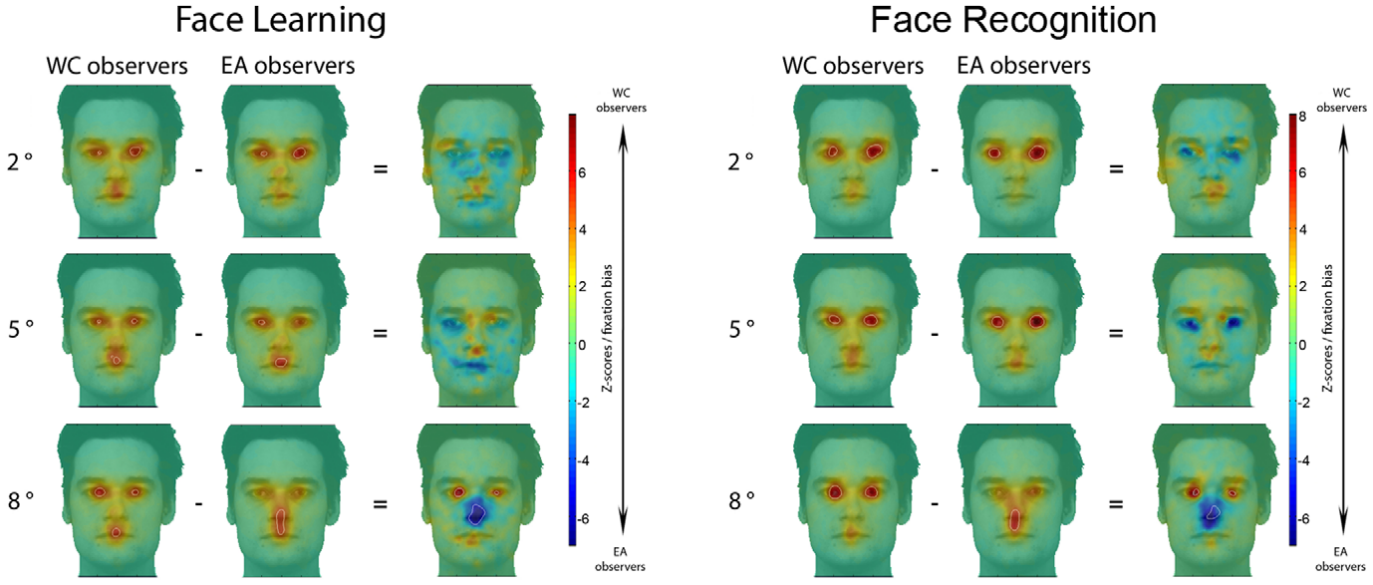
Fixation maps of Western Caucasian (WC) and East Asian (EA) observers for each *Spotlight* condition during WC and EA face learning (*left*) and recognition (*right*). Fixation biases for WC (red) and EA (blue) observers are highlighted by subtracting WC and the EA Z-scored group fixation maps. Areas fixated above chance are delimited by white borders. Note the shift of eye movement fixations in the EA observers in the 8° *Spotlight* condition for both tasks.

**Figure 5:**
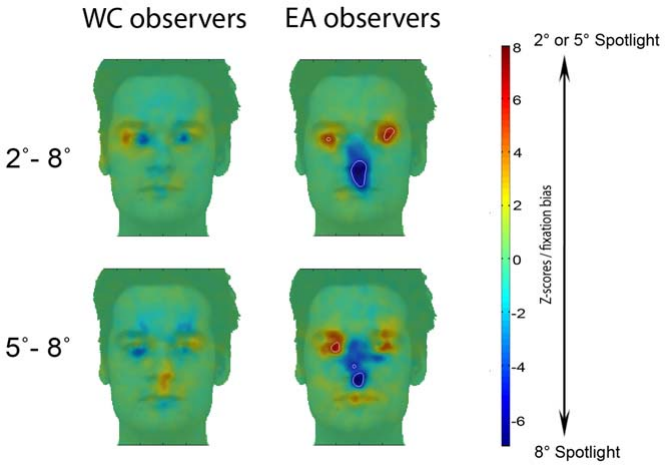
Fixation biases specific to the 8° *Spotlight*. *Top left: differential* fixation maps computed by subtracting the fixation map for Western Caucasian (WC) observers obtained in the 2°*Spotlight* condition with the 8° *Spotlight*. *Bottom left:* differential fixation maps computed by subtracting the fixation map for Western Caucasian (WC) observers obtained in the 5°*Spotlight* condition with the 8° *Spotlight*. *Top and Bottom right:* report the same comparisons, in their respective conditions, for the East Asian (EA) observers. Note that significant differences were only observed for East Asian observers, which deployed a central fixation bias only in the 8°*Spotlight* condition and focused on the eye region in the 2° and 5° constrained *Spotlight* conditions.

**Figure 6:**
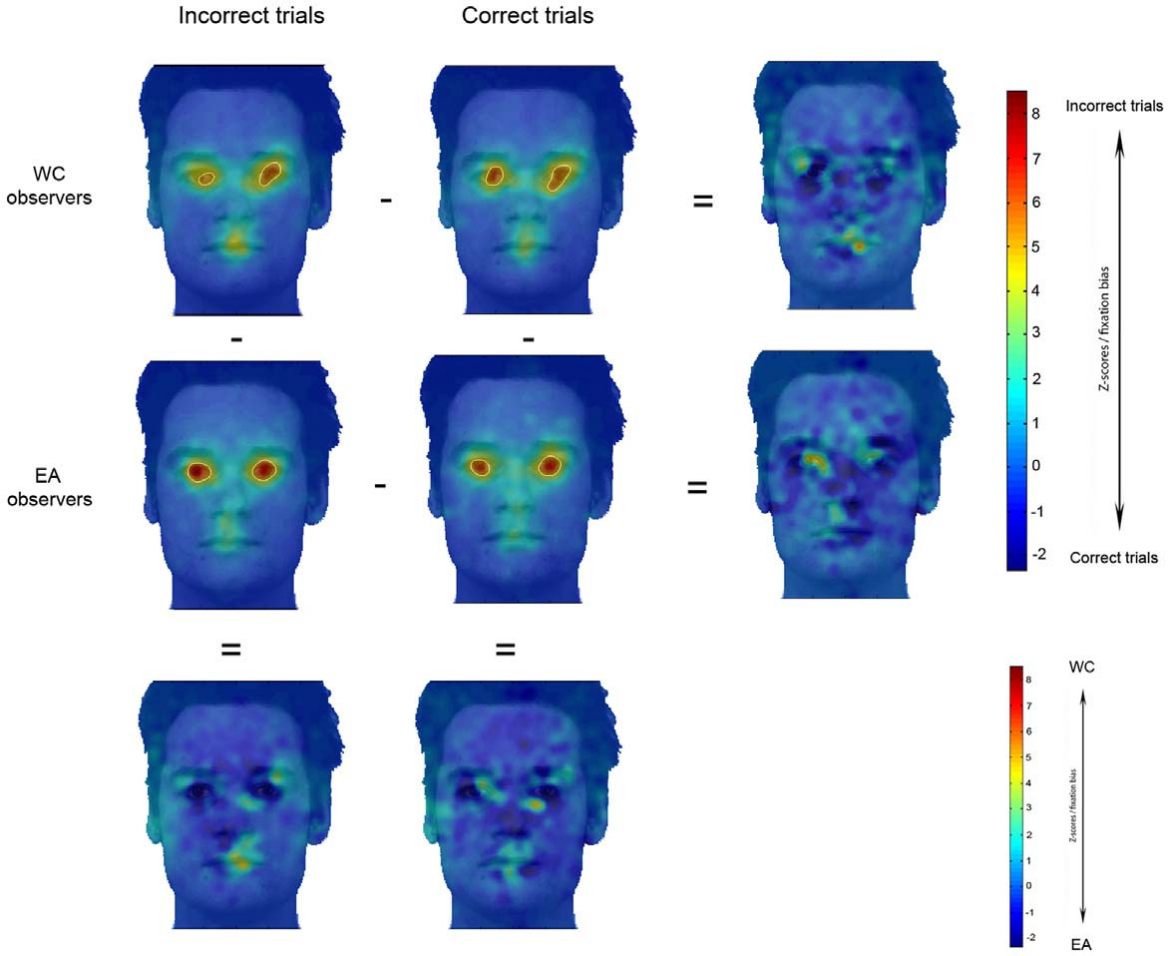
Fixation maps of Western Caucasian (WC) and East Asian (EA) observers for the 2° *Spotlight* condition during face recognition for incorrect and correct trials. Areas fixated above chance are delimited by white borders. Observers significantly fixated more on the eye region than the rest of the face regardless their accuracy (about 28 trials per condition); on those maps the darker blue represents 0. *Right*: Note the absence of significant differences in the fixation strategies as a function of correct and incorrect trials in both groups of observers. *Bottom*: differential fixation biases for Western Caucasian (WC - red) and East Asian (EA - blue) observers are highlighted by subtracting the WC and the EA Z-scored group fixation maps for incorrect and correct trials respectively. No significant cultural differences were found for this comparison.

**Figure 7:**
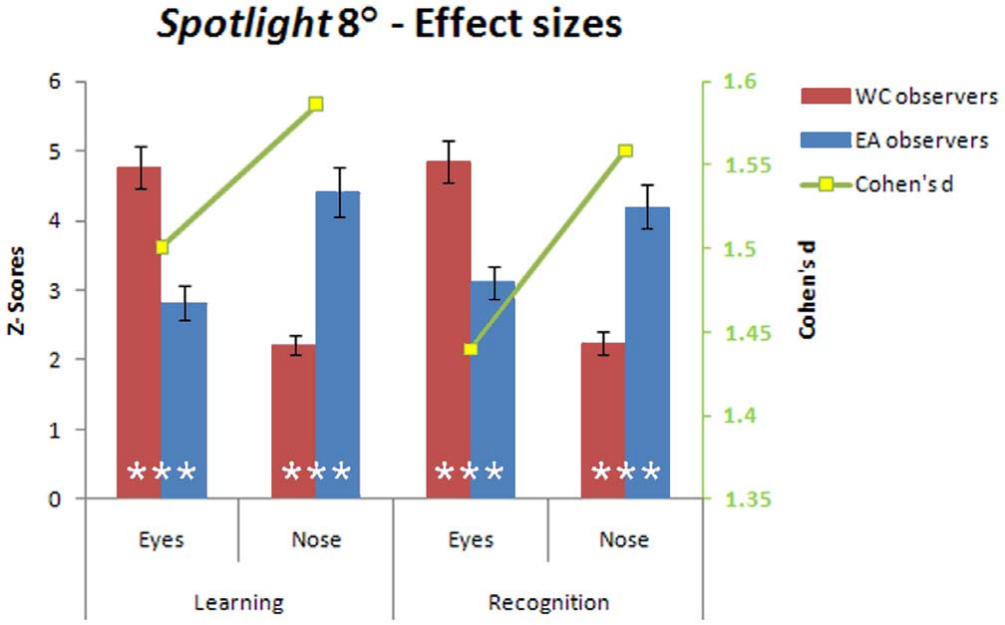
Average Z-score (left *y* axis) and *Cohen's d* effect size values (right *y* axis) for the eye and nose regions during face learning and recognition. WC  =  Western Caucasian; EA  =  East Asian. Error bars report standard errors of the mean. Significant differences between observers from different cultures for each of the facial features are reported at the bottom of the bars (***  = P<.001).

**Figure 8:**
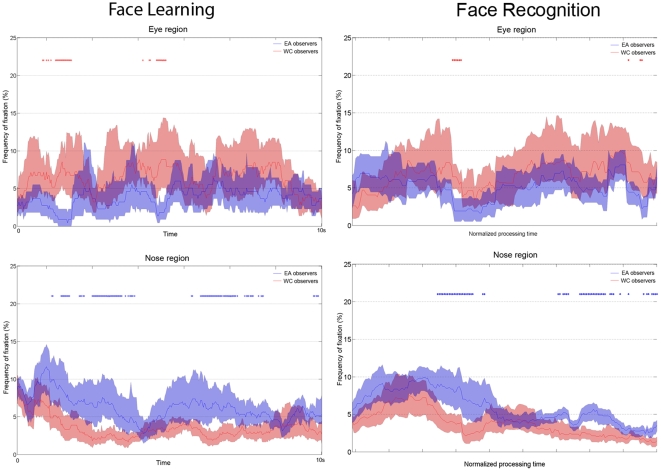
Time course of the frequency of fixations for the facial regions showing a cultural bias during face learning (left) and recognition (right). *Top*: the eye region; *bottom*: the nose region. Note that since the response times varied across trials within- and between-observers, the time was normalized for this condition only. Observers from different culture showed a larger number of fixations towards their respective preferred regions throughout the entire time course. Significant differences at each time point across the two groups of observers are reported by an *. Shaded areas report the 95% confidence interval (Western Caucasian – red; East Asian – blue).

These observations demonstrate that face processing does not arise from a universal series of perceptual events. Instead, the strategies employed to extract visual information from faces varies across cultures. We mainly attributed eye movement diversity in face processing to genuine systematic cultural perceptual differences observed between Westerners and Easterners. A growing body of literature (for a review see [10]) has reported systematic differences across cultures in a variety of perceptual tasks and paradigms: scene perception (e.g., [11]) and description (e.g., [12]), perceptual categorization (e.g., [13]) and eye movement for scene affordance [14]. All these studies converge into a similar pattern of results, revealing that distinct cultural mechanisms influence visual perception and categorization. Western cultures focus on salient objects or features and use *analytical* categorization rules to organize the environment. By contrast, Easterners focus more *globally* on relationships and similarities among objects when organizing the environment. Our previous eye movement data [2] suggest that Western Caucasian observers deploy an *analytical* perceptual strategy to integrate facial information by using feature-by-feature fixations, whereas East Asian observers focused on the region that is optimal and economical to integrate information *globally*: the center of the face (i.e., the nose region). The nose region is the most advantageous spatial position to capture facial feature information globally (see Figure 1 - 8° condition), since retinal cell density and visual resolution decrease steeply towards the peripheral visual field. One of the most prominent, despite debatable, position in the cultural framework posits the roots of the diversity in cultural perceptual strategies in the organization of the social systems in which people develop and live (for a review see [15], [16]). Western societies are *individualistic*, encouraging the development of individual goals, which would favor the perception of focal object in a context [17]. By contrast, Eastern societies are *collectivistic*, in which the group holds greater importance than the individual, favoring perception biases towards the relationship between objects (but see [18]).

Beyond the theoretical interpretations of our previous findings, it remains puzzling and necessary to explain *how* East Asian observers achieve face recognition by not focusing on the eyes. Indeed, the abundant literature on face recognition testing Western Caucasian observers in the recognition of Western Caucasian faces (e.g., [19], [20], [21]), with response classification techniques in normal healthy adults (e.g., [22], [23]) and brain damaged patients [24], and computational modeling (e.g., [25], [26]) have robustly shown in Westerners that the critical information for face recognition is located in the eyes and partially the mouth, but not the nose. These findings question the genuine use of information from the nose region in Easterner observers, or *at least* it remains to be unambiguously demonstrated that Easterners use *only* information from this region to recognize faces. Given that eye movements in natural viewing conditions do not provide unequivocal evidence on the measure of the visual information being used by observers [27], it is possible that information from the eyes is extracted without focal fixations. For instance, Kuhn and Tatler [28] have shown that people detecting successfully a magic trick do not necessarily fixate at the location on which the trick is taking place, demonstrating that a *natural* fixation does not straightforwardly translate information use. As a consequence, the question of whether humans universally use similar facial information to recognize faces, despite cultural variance in eye movements, remains unresolved.

To directly address this issue, we monitored eye movements of thirty Western Caucasian and thirty East Asian (i.e., Chinese) observers during the recognition of Western Caucasian and East Asian faces, using a gaze-contingent paradigm [29], a technique that has been extensively used in reading and scene perception literature (for a review see [30]). In gaze-contingent paradigms the stimulus display is continuously updated as a function of the observers' current gaze position. Therefore, the gaze-contingent technique is a powerful method to control for the visual information feeding the visual system and to isolate information use. In the language domain this method has been successfully used in natural reading to map out the perceptual span (moving window paradigm: e.g., [31], [32]), the nature of the extrafoveal information extracted during a fixation, for instance orthographic and phonological information (boundary paradigm: e.g., [29], [33], [34]) or the relative influence of attention versus acuity drop-off in the perceptual span (parafoveal magnification paradigm: e.g., [35]). Here, we adapted the gaze-contingent method to reveal the information *actively* used by observers to achieve face recognition. To this aim, we parametrically restricted the facial information available to the observers by using *‘Spotlights’* with Gaussian apertures dynamically centered on observers' fixations of 2° (foveal vision only), 5° and 8° (both expanding on extrafoveal vision). Crucially, in the 2° and 5° conditions, the *Spotlight* apertures covered an entire eye, but the eyes and the mouth were not visible when fixating the nose (Figure 1 and see also Supporting Movie S1). The 8° condition was the closest to natural viewing conditions; information from both eyes and the mouth was simultaneously available when fixating the nose. Observers from both cultures were randomly allocated to one of these conditions and learned two series of 14 of Western Caucasian and East Asian faces with neutral, happy or disgusted expressions presented for 10 seconds each. After a 30 second interval, observers indicated which of 28 faces (14 faces from the learning phase – 14 new faces) was familiar or not. The emotional expression of the familiar faces was changed between the learning and the recognition stage to avoid trivial image matching strategies. In order to rule out the possibility of an inherent bias in East Asian faces that would drive the typical central eye movement strategy used by Easterners (i.e., the presence of more information on the nose region in East Asian faces compared to Western Caucasian faces), we also carried out a pixel-based statistical analysis on the face images used in the experiment.

Our results show that when the eyes and the mouth were not visible when fixating the nose (constrained 2° and 5° *Spotlights* conditions – Figure 1), Westerners and Easterners observers rely on the very same information to recognize faces, by *actively* deploying triangular fixations mainly over the eyes and partially the mouth. The eye movement strategies of Westerners were not modulated by any of the *Spotlight* size apertures. By contrast, Easterners shifted their eye movement strategy in the condition closest to naturalistic viewing conditions (8° – Figure 1), with fixations landing at their preferred location: the center of the face. In line with previous findings [36], [37], [38], our analysis on the face images showed that the modulations in the fixation strategies deployed by observers from different cultures cannot be accounted by any obvious difference that would rely on differences in facial feature information between faces from different race.

## Results

### Behavior

The race of the faces did not interact with the culture of the observer in terms of accuracy (F(1, 54) = .49, P = .48) and response times (F(1, 54) = 2.71, P = .10), even in the condition with the largest aperture size: 8° (accuracy — F(1, 18) = 1.25, P = .27); response times — F(1, 18) = 1.75, P = .2). For this reason, we collapsed the data across faces (Figure 2).

Regardless of their culture, observers showed an increase of face recognition accuracy (F(2, 54) = 43.8, P<0.001) and faster response times (F(2, 54) = 15.63, P<0.001) as *Spotlight* aperture size increased. Western Caucasian and East Asian observers were as accurate (F(1, 54) = 2.18, P = .14) and fast (F(1, 54) = .014, P = .9) at recognizing faces. The interaction for accuracy (F(2, 54) = .6, P = .55) and response times (F(2, 54) = .58, P = .56) between the Culture of the observer and the *Spotlight* aperture size factors failed to reach significance.

### Number of fixations

We did not observe an interaction between the Culture of the observer and the *Spotlight* aperture sizes on the average number of fixations deployed during the learning (F(2, 54) = .99, P = .37) and the recognition stage (F(2, 54) = .13, P = .87) (Figure 3).

During learning, however, Western Caucasian observers used significantly more fixations (M = 28.8) than East Asian observers (M = 27) (F(1, 54) = 6.38, P = .014) and the number of fixations increased for both group of observers as *Spotlight* aperture size increased (F(2, 54) = 9.23, P<.001) (see Figure 1). During recognition, Western Caucasian observers (M = 18.6) performed significantly less fixations than East Asian observers (M = 20.6) (F(1, 54) = 4.06, P = .048). The number of fixations was not modulated by *Spotlight* aperture size within this condition (F(2, 54) = 1.27, P = .28).

### Eye movements

The novel result comes from the eye movement data: Figure 4 shows the regions (weighted by the fixation durations) significantly fixated above chance level with *Spotlights* of 2°, 5° and 8° during face learning and recognition respectively (Z*_crit_*>|4.25|, P<.05).

Western Caucasian observers systematically fixated the eye region and partially the mouth, regardless of *Spotlight* size. By contrast, East Asian observers' eye movement strategies were clearly altered by the information available. East Asians fixated the eye region (2° and 5°) and partially the mouth (5°) when the *Spotlight* constrained the available information, similarly to Westerners, as revealed by the absence of significant differences in the differential fixation maps (i.e., WC - EA Z-scored group fixation maps) for those conditions. Post-hoc analyses directly comparing the fixation maps during face learning in the 2° and 5° *Spotlight* conditions with the 8° condition for each cultural group separately, confirmed: i) the use of similar eye movement strategies in Westerners regardless of the *Spotlight* size aperture and ii) the presence of a genuine central fixation bias in Easterners uniquely with a Gaussian aperture of 8°, as well as fixations in the eye region in the 2° and 5° conditions (Figure 5 – similar results were observed during face recognition).

Analyses performed in the 2° condition for incorrect responses during face recognition (which roughly included a comparable number of trials and, therefore, a comparable variance with correct face recognition responses) show consistent information use from the eye region in both groups of observers (Figure 6, top and middle) and no significant differences in fixation strategies across observers from different cultures (Z*_crit_*>|4.25|, P<.05) (Figure 6, bottom).

Only trials leading to correct identification (hit and correct rejections) were taken into account for the face recognition stage in the 5° and 8° *Spotlight* conditions. When the eyes and the mouth regions were simultaneously accessible using extrafoveal vision (Figure 1 - 8°), East Asian observers shifted their fixations towards their preferred landing location during both face learning and recognition: the nose region (Figure 4 - 8° *Spotlight*). To determine the magnitude of the fixation biases across cultures, for each observer we extracted the average of the Z-scored values within the areas showing significant differences in the differential fixation maps (i.e., average Z-scored fixation duration of the eye and nose regions per observer). Then we carried out a two-way mixed design ANOVA on the averaged Z-score values with *Face regions* as a within-subject factor and *Culture of the observer* as a between-subjects factor. This statistical analysis revealed significant interactions for those factors in both conditions: learning (F(1, 18) = 40.08, P<.001, η*_p_* ^2^ = .69) and recognition (F(1, 18) = 33.56, P<.001, η*_p_* ^2^ = .65) (Figure 7).

Western Caucasian observers had significantly more fixations landing in the eye region, while East Asian observers had more fixations on the nose region, as revealed by independent two-tailed *t-tests* (P<.001). Cultural fixation biases on facial features were reliable and robust, as highlighted by the large magnitude of *Cohen's d* effect size values.

To finely track the appearance of such cultural fixation biases in processing faces, we computed the frequency of fixations over time for the fixations landing on the significant area around the eye and nose regions during face learning and recognition respectively. Western Caucasian observers showed a general bias towards the eye region over the entire time course during both tasks, with significant more fixations over the eyes compared to East Asian observers in particular time windows. By contrast, East Asian observers showed a general bias towards the nose region over the entire time course during both tasks, and significant morefixations over the nose compared to Western Caucasian observers in particular time windows (Figure 8).

**Figure 9:**
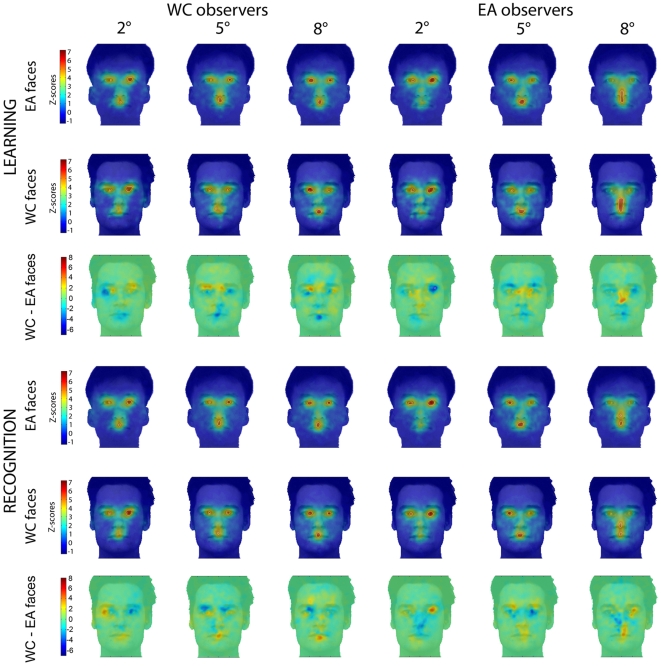
Caucasian (WC) and East Asian (EA) group fixation maps for learning and recognition trials. Face areas fixated with above chance frequency are delimited by white borders. As revealed by the lack of significant differences in the *differential* fixation maps (3^rd^ and 6^th^ row), fixation strategies of both cultural groups were consistent as a function of the race of the faces within their respective *Spotlight* conditions.

Regardless of the race of the input faces, fixation strategies deployed by both groups of observers during learning and recognition were consistent, as highlighted by the lack of significant difference in the differential fixation maps (Z*_crit_*>|4.25|, P<.05) (Figure 9).

### Face images

**Figure 10:**
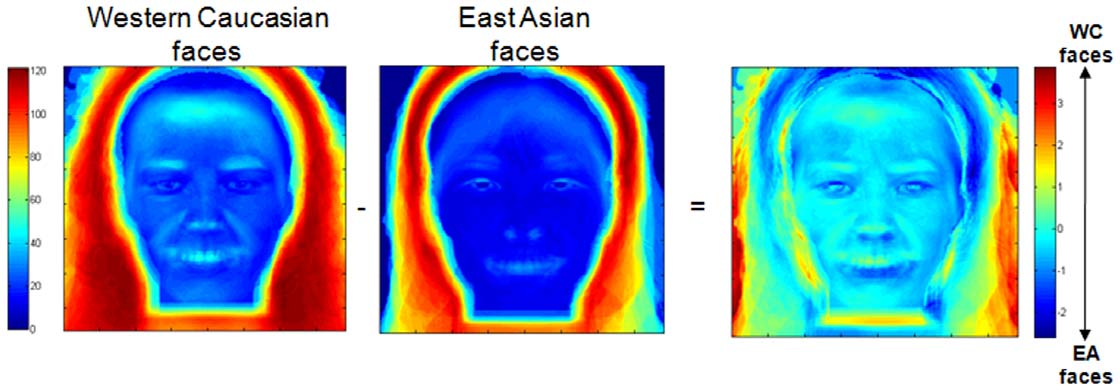
Standard deviation from the mean of the pixel values (gray level: 1-256) of the Western Caucasian (WC - *left*) and East Asian (EA - *middle*) faces used in the experiment. *Right*: Pixel information biases for WC (red) and EA (blue) faces. Note the absence of any significant differences in the pixel space across faces for faces from different culture (Z*_crit_*>|4.25|, P<.05), indicating the absence of evident diagnostic information for observers.

We calculated the standard deviation of the face images used in the present experiment, separately for each race. To identify region of the faces that would be significantly different across both populations, we subtracted these values and used a two-tailed *pixel* test on the differential fixation maps (Z*_crit_*>|4.25|, P<.05) (Figure 10).

This analysis failed to reveal any significant difference. There was no region in the faces from different race containing different information at the pixel level.

## Discussion

The gaze-contingent *Spotlight* technique showed that, independently of culture, observers rely on identical information to recognize faces. The *Spotlight* results with limited perceptual spans (2° and 5°) provide direct evidence on information use, as with limited extrafoveal information observers are constrained to *actively* focus on the diagnostic information required for face recognition. Notably, Westerners and Easterners showed similar eye movement scanpaths in these conditions, with extended fixations towards the eye region and partially the mouth, abolishing previously established cultural diversity in eye movements [2]. These findings are consistent with the triangular pattern of fixations reported in many previous eye movement studies using Western Caucasian observers [3], [4], [5], [6], [7], [8], [9] and overall they are fully in line with previous findings pointing to the eyes as a critical feature for face recognition (e.g., [19], [20], [21], [22], [23], [24], [25], [26]). Consistent with our previous observations in natural viewing conditions [2], East Asian observers changed their eye movement strategy and fixated the nose region in the 8° condition, since information from the eye and mouth region was extractable extrafoveally from this location.

Regardless of their culture, within their respective *Spotlight* conditions (Figure 9) observers did not change their viewing strategies for faces from different races, which is consistent with our previous eye movement findings [2]. Additionally, we did not find differences in terms of recognition performance across observers from different cultures, nor as a function of the race of the faces. As might be expected, artificially restricting information outside central vision impacted on the sensitivity of the face system and its performance, as the greater ability humans have in recognizing same- compared to other-race faces was abolished (e.g. [24], [39], [40], [41], [42]). In the present experiment we adopted an ecologically-valid approach by using different pictures of the same identify to tap into genuine face identification mechanisms (therefore ruling out potential face recognition mechanisms based on picture matching strategies). This experimental control represents a taxing constraint for the face system and might partially account for the present observations, as the vast majority of studies on the other-race effect have relied on the use of identical pictures to assess face recognition performance. It is worth noting that this observation is not critical for the purpose of the present study, as the eye movement data showed significant, consistent and robust fixation patterns for face recognition in both groups of observers. Restricting information available in extrafoveal vision also dramatically increased the number of fixations used by the observers to adapt to the present tasks at hand compared to natural viewing conditions (i.e., 5 fixations on average with natural vision during face recognition [2], compared to 18 here), with a series of first fixations directed towards locating regions of interest. It is worth noting, that most of those fixations were performed to precisely adjust fixations towards the location of interest and incidentally increased information sampling around the area. Importantly, those observations do not impact on the main aim of the present study, which was to isolate the information used by the observers to solve a face recognition task. Although both cultures were at chance level in the 2° condition, observers did not deploy random eye movement strategies: Westerners and Easterners consistently and significantly relied on the eyes to perform under those strong task constraints, even during erroneous face recognition (Figure 6). Western Caucasian and East Asian observers focused on similar facial areas (i.e., the eye and mouth regions) with a *Spotlight* of 5° to achieve above chance face recognition performance. The use of identical facial information in Western Caucasian and East Asian observers with constrained extrafoveal information (2° and 5°) suggests that the two groups of observers use the same facial information under natural viewing conditions (i.e., when the stimulus is a whole face) (Figure 4 and 5). Indeed, the *Spotlight* technique identifies precisely the information used by observers under constrained (i.e., foveated) and unconstrained (i.e., extra-foveated) conditions. Compared to response classification techniques (e.g., [22], [23]) the *Spotlight* technique offers the advantage of controlling and providing *active* dynamic information use with a very limited number of trials. However, the technique shares a comparable disadvantage with response classification techniques which relies in altering the information available compared to natural vision (i.e., full face).

Finally, our observations in the 8° condition report again a stark contrast between cultures. Observers from different cultures reached a comparable level of performance by deploying differential fixation patterns. WC observers persistently reproduced the well established triangular pattern of fixation over the eyes and the mouth [3], [4], [5], [6], [7], [8], [9]. Westerners tend to engage *analytic* strategies for processing the visual environment [16] and consequently their triangular facial feature-by-feature strategy [2] was not affected by *Spotlight* sizes. Uniquely in this 8° condition information from the eye region was extractable from the face center and EA observers shifted their fixation towards the nose region [2] (Figure 4, 5 and 7). This central location is optimal to integrate information *globally* [2] and satisfies with the constraints of the cultural perceptual tuning typical of East Asian observers [16]. Interestingly, such cultural diversity in facial feature fixation (i.e., WC towards the eye region; EA towards the nose region) is not restricted to a particular time period. The analysis of the time course of the frequency of facial feature fixations demonstrated that this oculo-motor behavior is more deeply rooted in the entire strategy deployed to process faces (Figure 8), confirming the robustness of this cultural perceptual bias.

It is worth noting that both WC and EA observers have been shown to perceive faces *holistically* (e.g., [41], [43], [44]). Therefore, eye movement scanpaths cannot rule out the possibility that WCs' feature-by-feature fixations might be used to construct a whole-face representation, while a similar representation might be elaborated from central fixations on the nose region by EA observers. We could also hypothesize that even if observers from different cultures use the same facial information, the spatial relations involving the nose region are more useful to elaborate such representations to East Asian than Western Caucasian observers. Note, that we intentionally decided to use here the term *global* − and not the term *holistic* as widely used in the cultural literature − to relate cultural differences in perception and eye movements by East Asian observers to avoid confusion with the term *holistic* used in the framework of face processing. Future studies are necessary to clarify *whether* and *how* such mechanisms are related (see [45]).

We previously suggested an alternative explanation for interpreting the central fixation strategy employed by EA observers [2], which relied on a social norm. Direct gazing at people during social interaction is considered to be rude in East Asian societies [46] and therefore this cultural force might have shaped the eye movement strategies used by East Asian observers. Interestingly, we recently investigated facial expressions of emotion categorization across cultures [47]. We found that Western Caucasian and East Asian observers deploy distinct, culture-specific fixation strategies to decode the same basic set of six facial expressions, plus neutral (using faces from both cultures). While Western Caucasian observers distribute their fixations evenly across the face (i.e. mainly to the eyes and mouth), East Asian observers persistently sample the eye region. Beside firstly showing cultural diversity in eye movements for expression categorization, these data show that East Asian do gaze on the eye region when it is necessary. Secondly, they demonstrate that the central fixation used by East Asian observers during face recognition relates to task *specific* perceptual mechanisms. In addition, we have recently shown that the central fixation deployed by East Asian observers expand on the recognition of non-facial visually homogenous objects [45], suggesting a more fundamental explanation may be required to clarify cultural diversity in eye movements. Future studies are necessary to clarify the effective use of extrafoveal information in Easterners, assess the extent to which their eye movement strategy is regulated by social norms and identify the cultural roots shaping their perceptual strategies in vision.

Behavioral [11], [12] and eye movement [14], [48] differences across people from different cultures have been also reported during scene affordance. Yet, this view has been recently challenged by a series of eye movement studies pointing out to a similar use of fixation strategies across observers from different cultures [49], [50], [51], widely opening a debate on this question. However, the eye movement sampling strategies for faces cannot be straightforwardly compared with previous studies in scene perception. For instance, the visual scenes are composed by many objects and cover a larger visual angle compared with faces (for a detailed discussion on this point, see [2]). But also, there is abundant evidence in the literature showing that human faces are a homogenous visual category that taps into cognitive and neural mechanisms that are distinct from those engaged in object or scene perception (for a review, see [52], [53]). The data reported here show universality (2° and 5°) and diversity (8°) for eye movements when people from different culture look at faces, but do not allow to conclude on the generalization of the fixation strategies that would be deployed for processing other types of visual information (i.e., scenes, objects, etc.).

It is also worth noting that the *Spotlight* technique we used here precisely identifies the information use under constrained (i.e., foveated) and unconstrained (i.e., extra-foveated) conditions. This *Spotlight* opens a wide range of possibilities to investigate several questions aiming to identify the precise nature of the visual information selected by eye movements and the visual scan strategy during face processing in normal and clinical populations (e.g., autistic, schizophrenic, prosopagnosic and agnosic patients), paving the way to dynamic information integration modeling.

Understanding how humans share basic perceptual mechanisms is as important as understanding human diversity. Face recognition is routinely and effortlessly achieved in every culture. Social experience and cultural factors shape the way humans think about the world [16] and regulate the *strategies* used to extract information from faces [2], [47]. However, these external forces do not modulate the *information* used to solve this critical biological feat: all human beings rely on the eye region to recognize conspecifics. Human eyes have evolved with a unique morphology among primate species, with a sclera surrounding the highly-contrasted iris [54]. Such a morphological structure maximizes the transmission of gaze [54] and expression signals [55], which are both critical for social interaction, and eye region information processing has dedicated neural bases (for a review, see [56]). Newborns (e.g., [57]) and neurons devoted to face processing (e.g., [58]) show also a notable tuning towards the upper part of high-contrasted non face stimuli [59], [60] – a visual contrast mapping into eye region. Monkeys deprived after birth of experience with faces also show remarkably preserved face recognition abilities, compared to other visual object recognition [61], suggesting the existence of an experience-independent ability for face processing. Despite these studies pointing to a particular status of the eye region in face recognition, there is no obvious diagnostic information in this region to allow face recognition, *at least* by using a *univariate* statistical analysis approach with the present face database (Figure 10). It could be possible that diagnostic information from the eyes are revealed by using a statistical threshold lower than the probability threshold criterion routinely used in scientific studies (i.e., P<.05) and by using a *multivariate* combinatory statistical information coding approach. Future studies are necessary to clarify the extent to which the evolutionary constraints of nature (which have shaped eyes' morphology to have an high visual contrast and optimally transmit social signals) or decoding experience in social signals from this region of the face (which is culture dependent) are responsible for the universal information use from the eyes during face recognition in humans.

## Methods

### Participants

Thirty Western Caucasian (11 males, 19 females) and thirty East Asian (11 males, 19 females) young adults (mean age 25.23 years and 23.9 years respectively) participated in this study. Fifteen East Asian participants were newly enrolled international students attending the University of Glasgow, being born in East Asia and arriving in a Western country (Glasgow, UK) for the first time. The average duration of residence in the UK upon testing was less than 6 months within the East Asian group from Glasgow. The 15 other East Asian participants were students at the Sun Yat-Sen University, Guangzhou, China. All participants had normal or corrected vision and were paid £6 or equivalent per hour for their participation. All participants gave written informed consent and the protocol was approved by the ethical committee of the Faculty of Information and Mathematical Sciences of the University of Glasgow and the ethical committee of the Department of Psychology of the University of Sun Yat-Sen.

### Materials

Stimuli were obtained from the KDEF [62] and AFID [63] databases and consisted of 56 East Asian and 56 Western Caucasian identities containing equal numbers of males and females. The images were 382×390 pixels in size, subtending 15.6° degrees of visual angle vertically and 15.3° degrees of visual angle horizontally, which represents the size of a real face (approximately 19 cm in height). Faces from the original databases were aligned by the authors on the eye and mouth positions; the images were rescaled to match those facial features position and normalized for luminance. Images were viewed at a distance of 70 cm, reflecting a natural distance during human interaction [64]. All images were cropped around the face to remove clothing and were devoid of distinctive features (scarf, jewelry, facial hair etc.). Faces were presented on a 800×600 pixel grey background displayed on a Dell P1130 19″ CRT monitor with a refresh rate of 170 Hz.

### Eye tracking

Eye movements were recorded at a sampling rate of 1000 Hz with the SR Research Desktop-Mount EyeLink 2K eyetracker (with a chin/forehead rest), which has an average gaze position error of about 0.25°, a spatial resolution of 0.01° and a linear output over the range of the monitor used. The dominant eye of each participant was determined by using a variation of the Porta test [65], [66], [67]. Observers were asked to extend one arm and align the pointer finger of the extended hand vertically with the corner of the room, with both eyes open. Then, observers were instructed to close one eye or the other alternately and reported which eye closure caused the largest alignment change. Only the dominant eye was tracked, although viewing was binocular. The experiment was implemented in Matlab (R2006a), using the Psychophysics (PTB-3) and EyeLink Toolbox extensions [68], [69]. Calibrations of eye fixations were conducted at the beginning of the experiment using a nine-point fixation procedure as implemented in the EyeLink API (see EyeLink Manual) and using Matlab software. Calibrations were then validated with the EyeLink software and repeated when necessary until the optimal calibration criterion was reached. At the beginning of each trial, participants were instructed to fixate a dot at the center of the screen to perform a drift correction. If the drift correction was more than 1°, a new calibration was launched to insure an optimal recording quality. The eyetracker, software and setting used in Glasgow and Sun Yat-Sen universities were identical.

The *Spotlight* was either 2°, 5° or 8° degrees of visual angle with a zero alpha value at the centre. The alpha value is the value of the alpha channel we used to create the Gaussian apertures combined with an image with as background to create the appearance of partial transparency. The alpha values increased with distance from center of gaze according to a Gaussian function and reached 1 (complete opacity) at the border of the aperture. The image outside the *Spotlight* was black and the background gray. The display contingent to gaze position updating required 1 ms to receive a sample from the eye-tracker, less than 7 ms to calculate the texture including the background and the Gaussian mask and between 0 and 6 ms to refresh the screen. Therefore, the display was updated depending on observers' looking position every 11 ms on average (between 8 and 14 ms), eliminating any impression of flickering for the observers (see also Supporting Movie S1).

In the 2° and 5° conditions, the *Spotlight* apertures covered an entire eye, but the eyes and the mouth were not visible when fixating the nose (Figure 1 and see also Supporting Movie S1). The 8° condition was the closest to natural viewing conditions; information from both eyes and the mouth was simultaneously available when fixating the nose.

### Procedure

Ten observers from each cultural group were randomly assigned to one of the three *Spotlight* conditions, with Gaussian apertures of 2°, 5° or 8° degrees. To ensure that observers would deploy a reliable strategy with such strong visual constraints, they performed the entire experiment with the same *Spotlight* aperture size. Participants started with a training session in order to familiarize them with the gaze contingent display. Then they were informed that they would be presented with a series of faces to learn and subsequently recognize. They were also informed that they would be given two face recognition blocks per race. In each block, observers were instructed to learn 14 face identities displaying randomly either neutral, happy or disgust expressions (7 females). After a 30 second pause, a series of 28 faces (14 faces from the learning phase – 14 new faces; 7 females) were presented and observers were instructed to indicate as quickly and as accurately as possible whether each face was familiar or not by pressing keys on the keyboard with the index of their left and right hand. Response times and accuracy were collected and analyzed for the purpose of the present experiment. Faces from each cultural group were presented in separate blocks, with the order of presentation for same- and other-race blocks being counterbalanced across observers. Response buttons were counterbalanced across participants.

Each trial started with the presentation of a central fixation cross. Then four crosses were presented, one in the middle of each of the four quadrants of the computer screen. These crosses allowed the experimenter to check that the calibration was still accurate, tolerating a maximum error 0.5 degrees of visual angle. A final central fixation cross, which served as a drift correction, was then followed by a face presented in a random location on the computer screen. If a fixation failed to land on any of the crosses during the firsts 2 seconds, a new calibration was started. In that way, we validated the calibration between each trial. Faces were presented in a black frame for 10 seconds duration in the learning phase and until the observer responded in the recognition phase. To prevent anticipatory strategies, images were randomly presented on different locations of the computer screen. Each face was subsequently followed by the 6 fixation crosses which preceded the next face stimulus.

### Data analyses

We implemented saccade detection in our Matlab routines analyzing eye movement, by using the same filter parameters as the EyeLink software: saccade velocity threshold  = 30°/sec; saccade acceleration threshold  = 4000°/sec. To detect a saccade, for each data sample, the parser thus computes velocity and acceleration and compares these to the velocity and acceleration thresholds. Sometimes, a large saccade is followed by a small corrective saccade or vice versa. As a result, two or more temporally (<20 ms) and spatially (<.30°) contiguous saccades could be merged. Additionally, a blink is defined as a period of saccade detector activity with the pupil data missing for three or more samples in a sequence. A fixation event is defined as any period that is not a blink or saccade.

Fixation distribution maps were extracted individually for Western Caucasian and East Asian observers and face race, for the learning and recognition tasks separately. The data from East Asian observers from China and from those newly arrived in Glasgow were analyzed separately. A two-tailed *Pixel test* (Z*_crit_* |4.25|; *p*<.05 – see below for statistical details) showed no differences across both groups of East Asian observers (see Supporting Figure S1). Therefore the data from both groups of East Asian observers were collapsed together. The fixation maps were computed by summing, across all (correct) trials, the fixation location coordinates (*x, y*) across time. This procedure directly weights the importance of a fixation as a function of its duration, thereby representing the time spent fixating a particular location. Since more than one pixel is processed during a fixation, we smoothed the resulting fixation distributions with a Gaussian kernel with a sigma of 10 pixels. Then, the fixation maps of all the observers belonging to the same cultural group were summed together separately for each face condition, resulting in group fixation maps.

We then Z-scored the resulting group fixation maps by assuming identical Western Caucasian and East Asian eye movement distributions for a particular face race as the null hypothesis. Consequently, we pooled the fixation distributions of observers for both groups and used the mean and the standard deviation for Western Caucasian and East Asian faces to separately normalize the data. To clearly reveal the difference of fixation patterns across observers of different cultures, we subtracted the group fixation maps of the East Asian observers from the group Western Caucasian and we Z-scored the resulting distribution. To establish significance, we used a robust statistical approach correcting for multiple comparisons in the fixation map space, by applying a one-tailed *Pixel test* [70] (Z*_crit_*>4.64; *p*<.05) for the *group* fixation maps and a two-tailed *Pixel test* (Z*_crit_* |4.25|; *p*<.05) on the *differential* fixation maps. Finally, for each condition we extracted the average Z-score values for each observer individually, within each region of interest showing significance in the *differential* fixation maps. *Cohen's d* effect sizes [71] and partial eta squares were calculated from a two-way mixed design ANOVAs on the average Z-scores with the Region of the face and the Culture of the observer as factors carried out for the learning and recognition conditions separately.

To isolate the time course of fixations landing in regions showing a cultural bias, we computed the frequency of fixations over time during face learning and recognition. The frequency was extracted at each time point sample and was normalized by the area covered by the regions of interest. The regions of interest were defined by selecting all the pixels falling within areas showing significant differences across cultures (i.e., nearby the eye region for WC observers; nearby the nose region for EA observers – see Figure 4). Given the rather heterogeneous, asymmetrical nature of the distributions of frequency of fixations over time, we also carried out percentile bootstrap analyses. We sampled observers with replacement, computing the mean frequency across participants independently for each condition. This process was repeated 5000 times, leading to a distribution of bootstrapped estimates of the mean frequency for each group of observers, averaged across subjects. Then the 95% percent confidence interval was computed (alpha  = 0.05). Finally, the difference between the two sample means was considered significant if the 95% confidence interval did not include zero. Note that this bootstrap technique, relying on an estimation of H1, tends to have more power than other robust methods like permutation tests and related bootstrap methods that evaluate the null hypothesis H0.

Finally, to identify whether eye movement strategies were modulated by the race of the faces, we computed a *differential* fixation map, by subtracting the eye movement patterns for Western Caucasian and East Asian faces in both groups of observers. Significance was established by using a two-tailed *Pixel test* (Z*_crit_* |4.25|; *p*<.05).

### Image analyses

In order to assess for the presence of obvious facial diagnostic information that would be inherently present in faces from different race, we carried out a statistical analysis in *pixel* space of the faces images we used. We averaged the grey level values (1-256) of all Western Caucasian and East Asian faces separately and calculated the average and standard deviation of the images. The standard deviation from the mean would represent the information available to discriminate across face exemplars, as this formally contains the information (*pixels*) that differs across exemplars (the average being the common information). Thus, we calculated a differential map across faces from different race, by subtracting the standard deviation of Western Caucasian face images from the standard deviation of East Asian face images. To establish significance, we used a two-tailed *Pixel test* (Z*_crit_* |4.25|; *p*<.05).

## Supporting Information

Figure S1 Fixation maps for the EA participants tested in Glasgow and those tested in China with the different *Spotlight* apertures conditions. No significant difference was found in the eye movement strategies deployed by these two groups of East Asian observers, so the data were collapsed together. Note, that these data also show that short term experience in a Western country does not modulate eye movements for faces in Easterners.
(5.39 MB TIF)

Movie S1 QuickTime™ movie of the eye movement strategy deployed by a Western Caucasian observer during the 10 seconds face learning with a 5° *Spotlight* aperture size.
(1.98 MB MOV)
