# Supplementary figures and images for: Putting Culture Under the ‘Spotlight’ Reveals Universal Information Use for Face Recognition
Source: PLoS One. 2010 Mar 18;5(3):e9708. doi: 10.1371/journal.pone.0009708 (PMC2841167; doi:10.1371/journal.pone.0009708)

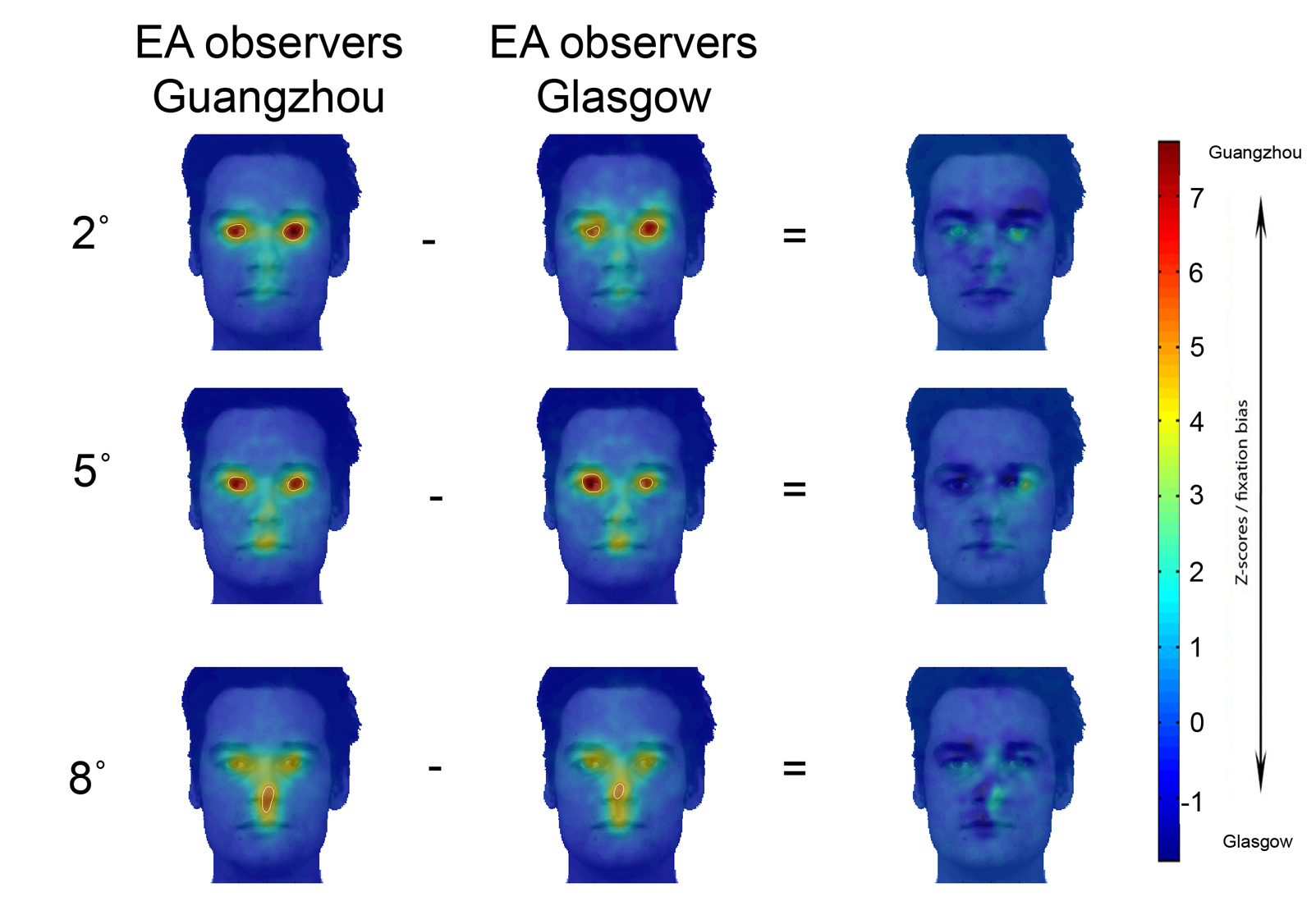

Supplement: Figure S1 — Fixation maps for the EA participants tested in Glasgow and those tested in China with the different Spotlight apertures conditions. No significant difference was found in the eye movement strategies deployed by these two groups of East Asian observers, so the data were collapsed together. Note, that these data also show that short term experience in a Western country does not modulate eye movements for faces in Easterners. (5.39 MB TIF) [file pone.0009708.s001.tif]
